# MtgA Deletion-Triggered Cell Enlargement of *Escherichia coli* for Enhanced Intracellular Polyester Accumulation

**DOI:** 10.1371/journal.pone.0125163

**Published:** 2015-06-03

**Authors:** Ryosuke Kadoya, Ken’ichiro Matsumoto, Toshihiko Ooi, Seiichi Taguchi

**Affiliations:** 1 Division of Biotechnology and Macromolecular Chemistry, Graduate School of Engineering, Hokkaido University, Sapporo, Hokkaido, Japan; 2 CREST, Japan Science and Technology Agency, Kawaguchi, Saitama, Japan; Institut Pasteur Paris, FRANCE

## Abstract

Bacterial polyester polyhydroxyalkanoates (PHAs) have been produced in engineered *Escherichia coli*, which turned into an efficient and versatile platform by applying metabolic and enzyme engineering approaches. The present study aimed at drawing out the latent potential of this organism using genome-wide mutagenesis. To meet this goal, a transposon-based mutagenesis was carried out on *E*. *coli*, which was transformed to produce poly(lactate-*co*-3-hydroxybutyrate) from glucose. A high-throughput screening of polymer-accumulating cells on Nile red-containing plates isolated one mutant that produced 1.8-fold higher quantity of polymer without severe disadvantages in the cell growth and monomer composition of the polymer. The transposon was inserted into the locus within the gene encoding MtgA that takes part, as a non-lethal component, in the formation of the peptidoglycan backbone. Accordingly, the *mtgA*-deleted strain *E*. *coli* JW3175, which was a derivate of superior PHA-producing strain BW25113, was examined for polymer production, and exhibited an enhanced accumulation of the polymer (7.0 g/l) compared to the control (5.2 g/l). Interestingly, an enlargement in cell width associated with polymer accumulation was observed in this strain, resulting in a 1.6-fold greater polymer accumulation per cell compared to the control. This result suggests that the increase in volumetric capacity for accumulating intracellular material contributed to the enhanced polymer production. The *mtgA* deletion should be combined with conventional engineering approaches, and thus, is a promising strategy for improved production of intracellularly accumulated biopolymers.

## Introduction

Polyhydroxyalkanoates (PHAs) are bacterial polyesters that can be developed as commodity plastic materials and also applicable for environmental and biochemical applications [1–4]. The material properties of PHAs are governed by their monomer composition, molecular weight and copolymer microstructure [5]. In addition, the efficient conversion of inexpensive and renewable feedstock into PHA results in value-added products that are competitive with their petroleum counterparts [6]. The primary aims of the metabolic engineering of PHA, therefore, include controlling the different factors that determine these polymer properties and optimizing yield. In this regard, a recombinant *Escherichia coli* system, which incorporates natural and engineered PHA biosynthetic pathways, is useful for achieving high-yield production of various tailor-made PHAs [7–9]. In addition, unlike many natural PHA produces, in which PHA accumulation is induced under the nitrogen and/or phosphate limited conditions, recombinant *E*. *coli* is able to produce PHAs under the nutrient rich condition probably because *E*. *coli* possesses no regulators controlling PHA biosynthesis.

To date, many rationally designed approaches have been examined to increase a flux toward PHA, such as reinforcement in the activities of PHA biosynthetic enzymes by means of gene dosage [10–12], and enzyme engineering [13–16]. In addition, disruption of competing pathways was also effective to improve the polymer production [17]. On the other hand, optimization of fermentation parameters such as pH, aeration rate etc, was useful to achieve high cell density and production per unit time [18,19]. These strategies for the metabolic and fermentation engineering of PHA producers were implemented either individually or in combination.

For further improvement in polymer production, we have explored positive factor(s) indirectly contributing to improved polymer production that is unable to be predicted based on rational approaches. Previously we evaluated the effect of deletion of four non-essential sigma factors, which is a global regulator governing the transcription of over 100 genes, of *E*. *coli* on the production of lactate-based polyester poly(lactate-*co*-3-hydroxybutyrate) [P(LA-*co*-3HB)], an engineered PHA with semitransparent and flexible properties (for detail, see [20]). This experiment aimed at exploring a global gene suppression that eventually increased polymer production. As a result, the *rpoN* deletion was found to improve P(LA-*co*-3HB) production in recombinant *E*. *coli* [21]. This result suggested the potential of this organism to be modified for enhanced polymer production. Therefore, in this study, we designed a transposon-based genome-wide mutagenesis of P(LA-*co*-3HB)-producing *E*. *coli* and high-throughput screening of highly-accumulating mutants. This approach expectedly isolates beneficial single gene knockouts that increased polymer production among all non-lethal gene disruptants. Indeed, one strain bearing a disruption of MtgA, which is involved in formation of the peptidoglycan strand [22–24], was isolated as a positive mutant. Interestingly, the selected mutant exhibited phenotype of enlarged cell size, which was associated with polymer accumulation. To the best of our knowledge, this is the first case of the single gene deletion that induced both cell size enlargement (a so-called *fat cell*), and enhanced polymer production.

## Materials and Methods

### Plasmids, strains, and growth conditions

**Fig 1:**
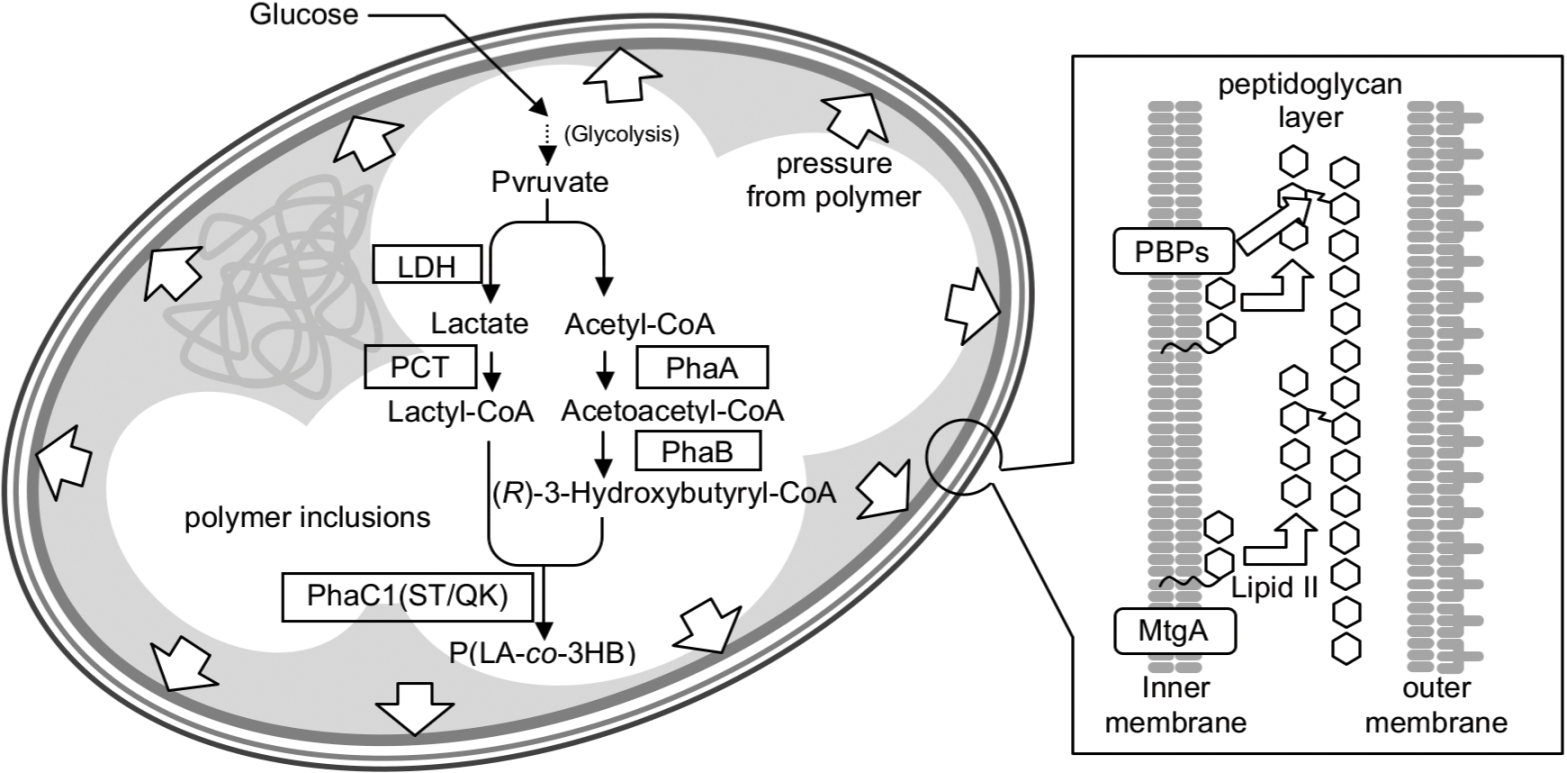
Model of polymer accumulation in fat *E*. *coli* cell with *mtgA* deletion. MtgA is a dispensable monofunctional glycosyltransferase catalyzing the polymerization of lipid II for the extension of glycan strands but not cross-linking. Penicillin-binding proteins (PBPs), which are bifunctional transpeptidases-transglycosylases and monofunctional transpeptidases, play a central role in the peptidoglycan formation. The *mtgA* deletion had no obvious effect on cell morphology without polymer accumulation, but generated a fat cell phenotype with polymer production. P(LA-*co*-3HB) production from glucose in *E*. *coli* was achieved by expressing four heterologous enzymes; β-ketothiolase (PhaA), acetoacetyl-CoA reductase (PhaB), propionyl-CoA transferase (PCT) and lactate-polymerizing engineered polyhydroxyalkanoate synthase [PhaC1(ST/QK)]. D-Lactate dehydrogenase (LDH) is an intrinsic enzyme. The polymer synthesis may elevate turgor pressure, which expands the cell to form the fat-cell and allowed to accumulate the additional amount of polymer.

*E*. *coli* strains used in this study are listed in Table 1. The expression vector pTV118N*pctphaC1*_Ps_(ST/QK)*AB*, which harbors genes encoding propionyl-CoA transferase from *Megasphaera elsdenii* (*pct*) [25], engineered PHA synthase with LA-polymerizing activity [*phaC1*_*Ps*_(ST/QK)] from *Pseudomonas* sp. 61–3 [26] and 3HB-CoA supplying enzymes β-ketothiolase and acetoacetyl-CoA reductase (*phaA*, and *phaB*) from *Ralstonia eutropha* [27], was used for P(LA-*co*-3HB) production [16,28]. The synthetic pathways of P(LA-*co*-3HB) was shown in Fig 1. pTS52 was used as a helper plasmid for conjugation [29]. For polymer production, recombinant *E*. *coli* harboring pTV118N*pctphaC1*_Ps_(ST/QK)*AB* were grown on 1.7 ml LB medium containing 20 g/l glucose and 10 mM calcium pantothenate at 30°C for 48 h with reciprocal shaking at 180 rpm. Ampicillin (Amp; 100 μg/ml), kanamycin (Km; 25 μg/ml), and chloramphenicol (Cm; 25 μg/ml) were added when needed. pCA24N-*mtgA* was obtained from ASKA clone [30].

**Table 1:** *E*. *coli* strains used in this study.

Strains	Genotype	Purpose	Reference
S17-1	MG1655 RP4-2-*tc*::[ΔMu1::*aac*(3)IV-Δ*aphA*-Δ*nic*35-Δ*Mu*2::*zeo*] Δ*dapA*::(*erm-pir*) Δ*recA*	Transposon mutagenesis of JM109	[48,49]
JM109	*endA*1 *glnV*44 *thi*-1 *relA*1 *gyrA*96 *recA*1 *mcrB*+ Δ(*lac-proAB*) e14- [F' *traD*36 *proAB*+ *lacIq lacZ*ΔM15] *hsdR*17(rK-mK+)	Host strain of transposon mutagenesis	[49]
JM109 C21	JM109 Δ*mtgA*∷Tn5	Selected mutant of JM109 as a enhanced polymer producer	this study
BW25113	Δ(*araD-araB*)567 Δ*lacZ*4787(::*rrnB*-3) *lacIp*- 4000(*lacIq*) λ- *rph*-1 Δ(*rhaD-rhaB*)568 *hsdR*514	Parent strain of Keio collection mutants	[38]
JW3175	Δ*mtgA*::FRT-*kan*-FRT	A mutant in Keio collection	[38]

### Construction of mutant library of *E*. *coli* JM109 and screening of the positive mutants

*E*. *coli* S17-1 λ-pir carrying pUTmini-Tn5 Km (Amp^r^, Biomedical, Seville, Spain [31]) was used for the conjugative transfer of the mini-Tn5 transposon (Km^r^) to recombinant *E*. *coli* JM109 harboring pTV118N*pctphaC1*_Ps_(ST/QK)*AB* (Amp^r^) and pST52 (Cm^r^). The S17-1 and JM109 cells were conjugated on an LB agar plate at 30°C for 16 h. The cells were suspended in 10 mM MgSO_4_ and grown on LB plates containing Cm, Km and Amp. Cm was used to get rid of S17-1 cells. Km was used to select transposon-inserted JM109 cells. Amp was used to maintain pTV118N*pctphaC1*_Ps_(ST/QK)*AB*. The plates were incubated at 30°C for 16 h. The transposon-inserted JM109 harboring pTV118N*pctphaC1*_Ps_(ST/QK)*AB* was screened on LB plates containing 2% glucose, Amp, Km and 0.5 μg/ml Nile red (Sigma-Aldrich). Colonies emitting strong fluorescence were chosen as candidates under a transilluminator and subjected to HPLC analysis for determining polymer production as described previously [32]. In brief, cells were directly treated with concentrated sulfuric acid at 100°C to convert polyester into unsaturated carbonic acids, which were measured using UV detector at 210 nm [33,34]. The concentration of glucose in the supernatant was determined by HPLC equipped with a refractive index detector, as previously described [35].

### Identification of transposon insertion site

The transposon insertion site was identified using inverse PCR method. Chromosomal DNA of *E*. *coli* was digested with *Pst*I, self-ligated [36] and amplified by PCR using a pair of primers: 5′-AAGGTGATCCGGTGGATGAC-3′ and 5′-CAATCGGCTGCTCTGATGCCGC-3′, which annealed to the Km resistance gene in the transposon [37]. The amplified fragment was sequenced to identify the transposon insertion site in *E*. *coli* chromosome.

### Measurement of cell density using flow cytometry

The volumetric cell density (cells/l) was measured by flow cytometry using a SH800 cell sorter (SONY). Cells grown under aforementioned conditions were harvested at 48 h (OD_600_ between 20 and 25) and 10-fold diluted sample with water was analyzed. The flow rate was set to 11 μl/min (pressure 2). All FSC (forward scatter) and SSC (side scatter) images were recorded using SH800 software (SONY).

### Determination of cell size

Cells were grown on polymer-producing conditions and harvested at 48 h. The cell images were captured using a microscopy BZ-X700 (Keyence). On the digital images, the length of polar axis (*x*) and diameter (*y*) of 150–200 cells for each condition were measured using ImageJ software (http://rsb.info.nih.gov/ij/index.html). Based on the " lemon-shaped " morphology of polymer accumulating cells, the cell volume (*V*) was approximated by that of oval sphere, and thus, calculated using the following formula (1).

$$V=\frac{4}{3}\pi \cdot \frac{x}{2}\cdot {\left(\frac{y}{2}\right)}^{2}$$ (1)

## Results and Discussion

### Transposon mutagenesis and screening of the highly polymer-producing mutant

A mutant library of the *E*. *coli* JM109 strain producing P(LA-*co*-3HB) was prepared by using the transposon mini-Tn5. A high-throughput screening of polymer-accumulating cells was performed by means of plate assay using Nile red-containing agar plates. The dye-staining allowed us to readily screen the candidates with higher polymer production than that of original recombinant (parent control). The strain JM109 was used as a host because the cells were efficiently stained with Nile red and our group has developed several in vitro evolved enzymes involved in PHA biosynthesis using the screening method [15]. Among approximately 10,000 colonies, 100 colonies were chosen as the first stage candidates. The polymer production in the candidates was determined using HPLC, and eventually, one mutant C21, which exhibited an enhanced polymer accumulation, was isolated. The mutant C21 produced 5.1 g/l polymer compared to the parent recombinant (2.9 g/l, S1 Table).

### The deletion of *mtgA* gene contributed to the enhanced polymer production

Nucleotide sequence analysis of chromosomal DNA of C21 revealed that the transposon was inserted into the *mtgA* gene. Accordingly, this knowledge was applied to a superior P(LA-*co*-3HB)-producing strain *E*. *coli* BW25113, which has been extensively used for the polymer production [17,32]. An *mtgA*-deleted derivative of BW25113, *E*. *coli* JW3175, was obtained from Keio collection [38,39]. Recombinant JW3175 and BW25113 harboring pTV118N*pctphaC1*_Ps_(ST/QK)*AB* (referred as rJW and parent recombinant, respectively) were grown on glucose to induce polymer accumulation (Table 2). rJW produced increased amount of P(LA-*co*-3HB) (7.0 g/l) compared to the parent recombinant (5.2 g/l) as observed in C21. To confirm a contribution of *mtgA* deletion to the enhanced polymer production, a complementary experiment of rJW was carried out by heterologous expression of *mtgA* gene [30]. The complementation recovered the phenotype the parent recombinant (Table 2). Thus, it was concluded that the deletion of the *mtgA* gene led to an increase in P(LA-*co*-3HB) production in *E*. *coli*. The *mtgA*-deletion had little effect on the LA/3HB ratio in the copolymer (Table 2).

**Table 2:** P(LA-*co*-3HB) production in *mtgA*-deleted and complemented strains.

Genotype	Plasmid	Cell dry weight (g/l)	True cell weight (g/l)	Polymer production (g/l)		
				Total	LA	3HB
Wild type	pTV118N*pct phaC1*_Ps_(ST/QK)*AB*	9.2 ± 0.2	4.1 ± 0.3	5.2 ± 0.1	0.8 ± 0.1	4.4 ± 0.1
Δ*mtgA*	pTV118N*pct phaC1*_Ps_(ST/QK)*AB*	11.6 ± 1.0	4.6 ± 0.9	**7.0** ± **0.4**	1.0 ± 0.1	6.0 ± 0.4
Δ*mtgA* ^*a*^	pTV118N*pct phaC1*_Ps_(ST/QK)*AB* + pCA24N-*mtgA*	8.0 ± 0.7	3.2 ± 0.3	4.9 ± 0.3	0.9 ± 0.1	3.9 ± 0.3

*E*. *coli* BW25113 (wild type) and JW3175 (Δ*mtgA*) harboring pTV118N*pctphaC1*_*Ps*_(ST/QK)*AB* were grown on LB medium containing 20 g/l of glucose at 30°C for 48 h with reciprocal shaking at 180 rpm. The data represent the average ± standard deviation of three independent trials. pCA24N-*mtgA* bears the *mtgA* gene, which is expressed by *lac* promoter.

^a^ 100 μM IPTG was added.

**Fig 2:**
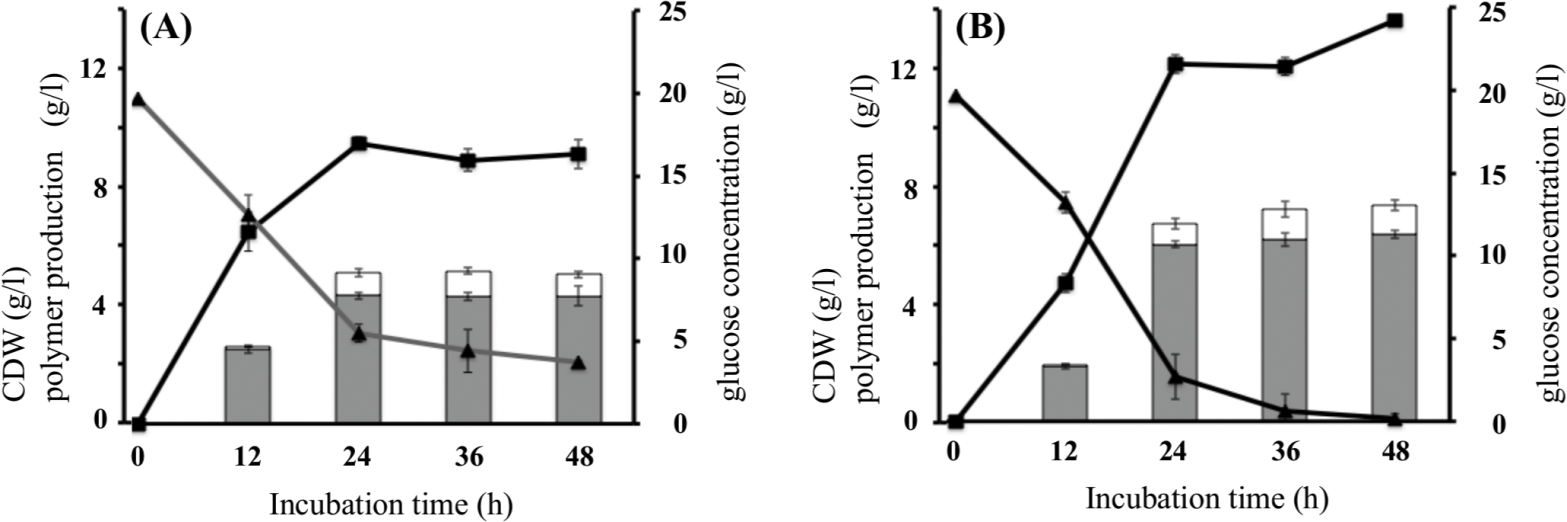
Time course of P(LA-*co*-3HB) production in the *mtgA*-deleted *E*. *coli*. *E*. *coli* BW25113 (wild type) (A) and JW3175 (Δ*mtgA*) (B) harboring pTV118N*pctphaC1*_*Ps*_(ST/QK)*AB* were grown on LB medium containing 20 g/l glucose. Triangle, glucose concentration in the medium. Square, cell dry weight. Gray bar, amount of 3HB unit in the polymer. White bar, amount of LA unit in the polymer. The data represent the average ± standard deviation of three independent trials. The cells were inoculated at time zero.

The time profile of polymer accumulation and glucose consumption are shown in Fig 2. At initial stage, rJW produced slightly less amount of polymer than parent recombinant, but at 24 h, achieved higher production. Notably rJW consumed glucose more rapidly than parent recombinant. The yield of the polymer from glucose in rJW (3.6 g/g) was slightly higher than the control (3.1 g/g). Therefore, *mtgA*-deletion increased both of the glucose consumption and conversion efficiency into polymer.

### Cell size enlargement caused by polymer accumulation in *mtgA*-deleted strain

The *mtgA* gene has been reported to encode a monofunctional peptidoglycan transglycosylase involved in the polymerization of lipid II molecules into glycan strands of peptidoglycans [40]. This fact prompted us to observe the cell morphology of the *mtgA*-deleted strain with polymer accumulation. Under the non polymer-producing conditions, as expected, JW3175 exhibited similar cell size to the parent strain BW25113 (Table 3). Thus, the *mtgA* deletion alone did not affect the cell morphology. On the contrary, under the polymer-producing conditions, rJW cells remarkably increased in size (1.4-fold) compared to the parent recombinant (Table 3). Interestingly, the *mtgA* deletion led to an increase in only the cell diameter but not length of polar axis, thus cells became *fat* rather than *tall*. Furthermore, the complemented strain of rJW exhibited cell size similar to the parent recombinant (data not shown), supporting that the *mtgA* deletion contributed to the cell enlargement. The transposon-inserted strain JM109 C21 exhibited similar cell morphology (data not shown).

**Table 3:** Correlation between polymer production and cell volume in recombinant *E*. *coli* with *mtgA* deletion.

Genotype	Plasmid	Cell density (l^-1^)	Single cell dry weight (g)	Cellular polymer content (wt%)	Polymer production in single cell (g)	Size of cell
							Polar axis (μm)	Diameter (μm)	Volume (μm^3^)
Wild-type	pTV118N	0.73×10^12^	2×10^–12^	N.D.	N.D.	2.26 ± 0.56	1.10 ± 0.12	1.43
Wild-type	pTV118NpctC1(STQK)AB	1.2×10^12^	8×10^–12^	56.8 ± 1.8	4.5×10^–12^	3.36 ± 0.82	1.15 ± 0.16	2.56
Δ*mtgA*	pTV118N	0.75×10^12^	2×10^–12^	N.D.	N.D.	2.49 ± 0.62	1.14 ± 0.23	1.69
Δ*mtgA*	pTV118NpctC1(STQK)AB	0.96×10^12^	12×10^–12^	60.9 ± 3.5	7.3×10^–12^	3.48 ± 0.84	1.42 ± 0.25	**3.67**

*E*. *coli* BW25113 (wild type) and JW3175 (Δ*mtgA*) harboring pTV118N*pctphaC1*_*Ps*_(ST/QK)*AB* were grown on LB medium at 30°C for 48 h. Cell density was measured using a flow cytometory. Cellular polymer content was defined as a ratio of polymer weight over total cell dry weight. The data represent the average ± standard deviation of three independent trials. The size of cells was determined using microscopic images of 150–200 cells. N.D., not detectable.

**Fig 3:**
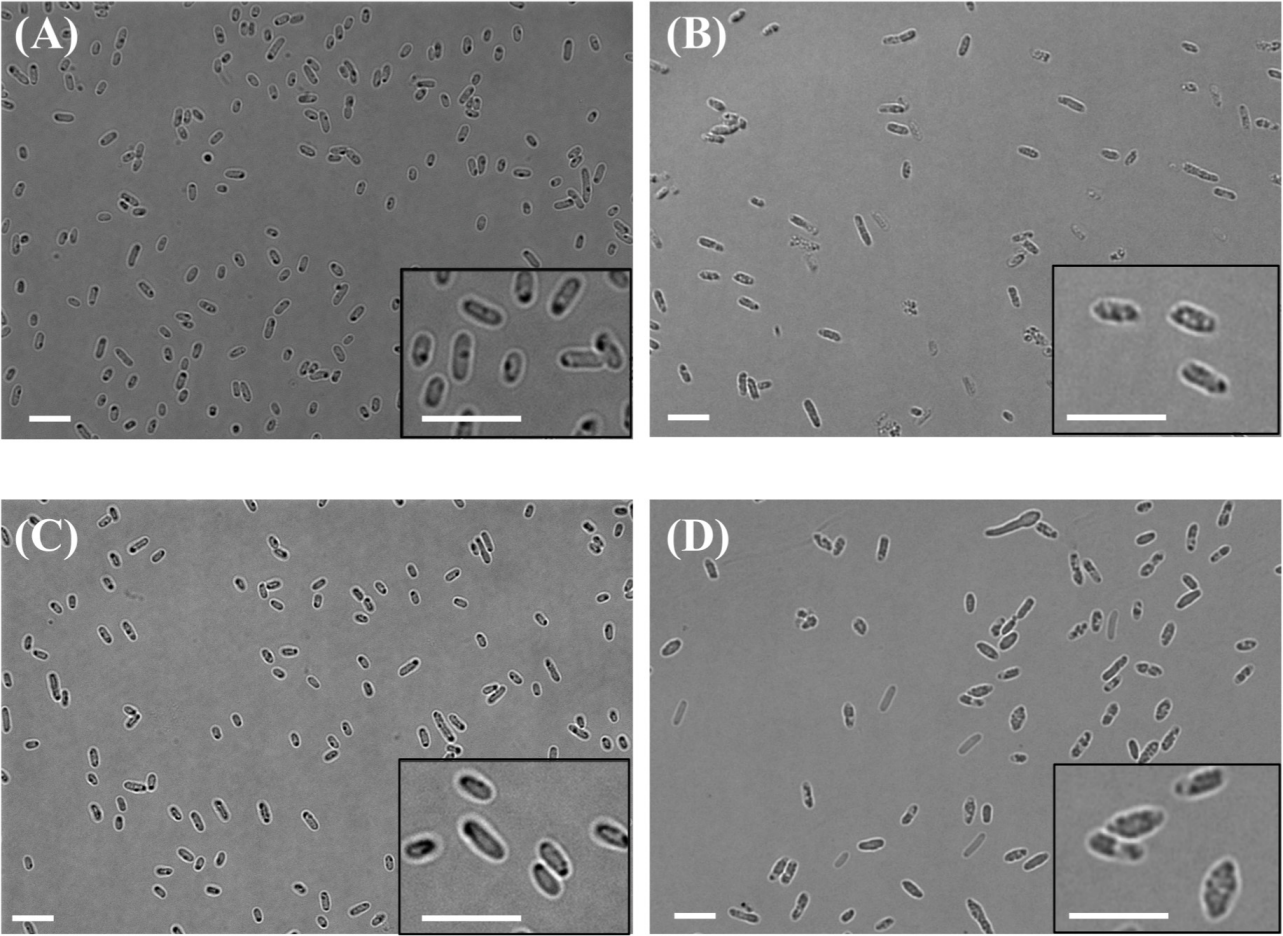
Effect on the cell volume exerted by polymer production in the wild-type and *mtgA*-deleted *E*. *coli*. The cells were grown on LB medium containing 2% glucose. *E*. *coli* BW25113 (wild-type) harboring pTV118N (empty vector) (A) and pTV118N*pctphaC1*_*Ps*_(ST/QK)*AB* (B). *E*. *coli* JW3725 (Δ*mtgA*) harboring pTV118N (C) and pTV118N*pctphaC1*_*Ps*_(ST/QK)*AB* (D). The cells harboring pTV118N did not produce detectable amount of polymer. Scale bars = 5 μm.

The cell morphology of rod-shaped bacteria such as *E*. *coli* is determined by a balance between elongation and septation of the cells, in which the peptidoglycan synthesis plays a central role. *E*. *coli* peptidoglycan is synthesized by multiple penicillin-binding proteins (PBPs), which are categorized into three classes; bifunctional transpeptidases-transglycosylases (class A), monofunctional transpeptidases (class B) and endopeptidases (class C) [40]. In the case of *E*. *coli*, deletion of class B PBP3 produced filamentous cells because the cells are unable to septate, whereas deletion of class B PBP2 resulted in an increase in the diameter of the cell [41]. These results suggest that the removal of cross-linking enzymes influenced the cell morphology. On the other hand, MtgA, a dispensable monofunctional transglycosylase, has been thought to play an auxiliary role in peptidoglycan synthesis. In fact, *mtgA* deletion alone exhibited no obvious effect on cell morphology (Fig 3) [42]. However, the result of present study showed that the cells lacking MtgA increased diameter presumably by outward force from the intracellularly synthesized polymer (Table 3), suggesting that MtgA does contribute to the peptidoglycan synthesis in *E*. *coli*. In addition, *mtgA* deletion did not induce filamentation of the cells, suggesting no critical effect on septation. This phenotype was contrast to the cell elongation induced by inhibition of FtsZ [43]. Currently, the three-dimensional structure of peptidoglycan remains elusive and there is ongoing argument on this issue [44–47], and therefore, an effect of *mtgA* deletion on the peptidoglycan structure also remains uncharacterized. The *mtgA* deletion may increase the flexibility of cell wall that allowed cells to expand in width and to accumulate extra amount of polymer.

### Quantitative analysis of the polymer production in fat cell

Volumetric polymer production (P) is determined using the following formula:

P(g/l)=Cell density(cells/l)×Single cell weight(g/cell)×Cellular polymer content(wt%)

In order to gain an advantage of the cell enlargement, the impact on cell growth is a primarily important factor. The growth of rJW was slightly slower than the control at the initial stage (Fig 3), but the decrease in the cell density (number of cells per volume. Cell density was measured using a flow cytometory.) of polymer-accumulating rJW at 48 h from parent recombinant was as small as 20% (Table 3), suggesting that there was no severe influence of *mtgA*-deletion on cell growth. In addition, the *mtgA*-deleted strain appeared robust and no cell lysis was observed (S1 Fig).

Another important value is the polymer production per cell. Based on the cell dry weight and cellular polymer content, the polymer production in a single cell of rJW was estimated to be 7.3 × 10^–12^ g/cell, which was 1.6-fold greater than the parent recombinant (4.5 × 10^–12^ g/cell). This result clearly indicated that the rJW *fat* cell possessed higher capacity of accumulating polymer. The increase in cell size should be necessarily accompanied with an increase in the amount (area) of cell membranes. In fact, the true cell weight (subtraction of polymer weight from total cell dry weight) of rJW was greater than parent recombinant when cells accumulated polymer (Table 3). However, the benefit of the increased accumulation capacity for PHA outweighed the additional consumption of carbon source for cell formation, and overall, the polymer production (g/l) and the polymer yield over glucose consumption (g/g) in rJW were increased. This fact conversely indicates that the size of intracellular space has been a limiting factor in the polymer accumulation in *E*. *coli*, and the *mtgA* deletion loosened the limitation.

In summary, P(LA-*co*-3HB) production in recombinant *E*. *coli* was elevated by disruption of the *mtgA* gene that led to a formation of *fat cell*. The same phenomenon was observed for P(3HB) production in *mtgA*-deleted strain (S2 Fig and S2 Table), indicating that the beneficial effect of *mtgA* deletion is not limited to P(LA-*co*-3HB) but should be applicable to wide range of intracellularly accumulated compounds. In addition, a synergy of combining *mtgA* deletion and conventional engineering approaches would be useful for further increasing polymer production.

## Supporting Information

S1 Fig Leakage of intracellular protein in the medium from *E*. *coli* BW25113 and JW3175 (Δ*mtgA*). Cells were grown on LB medium at 30°C for 48 h. Proteins in culture medium was concentrated with acetone and applied to SDS-PAGE. M, size marker. 1, BW25113. 2. JW3175. There was no significant difference in protein level in the medium, indicating that no cell lysis was promoted by *mtgA* deletion.
(EPS)

S2 Fig Effect on the cell volume exerted by P(3HB) production in the *mtgA*-deleted strains. *E*. *coli* BW25113 (wild-type) harboring pGEM*phaC1*_Ps_(ST/QK)*AB* grown on LB medium containing glucose (A). *E*. *coli* JW3725 (Δ*mtgA*) harboring pGEM*phaC1*_Ps_(ST/QK)*AB* grown on LB medium containing glucose (B). The cells produced P(3HB) under the presence of glucose. Scale bar = 5 μm.
(EPS)

S1 Table P(LA-*co*-3HB) production in *E*. *coli* JM109 and selected transposon mutant. All strains were grown in 1.7 ml of LB medium containing 20 g/l of glucose at 30°C for 48 h with reciprocal shaking at 180 rpm. The data represent the average ± standard deviation of three independent trials.
(DOCX)

S2 Table P(3HB) production in *mtgA*-deleted and complemented strains. *E*. *coli* BW25113 (wild type) and JW3175 (Δ*mtgA*) harboring pGEM*phaC1*_Ps_(ST/QK)*AB* [50] were grown on LB medium containing 20 g/l of glucose at 30°C for 48 h with reciprocal shaking at 180 rpm. The data represent the average ± standard deviation of three independent trials.
(DOCX)
