# Supplementary material for: MtgA Deletion-Triggered Cell Enlargement of Escherichia coli for Enhanced Intracellular Polyester Accumulation
Source: PLoS One. 2015 Jun 3;10(6):e0125163. doi: 10.1371/journal.pone.0125163 (PMC4454544; doi:10.1371/journal.pone.0125163)
Supplement: S1 Table — All strains were grown in 1.7 ml of LB medium containing 20 g/l of glucose at 30°C for 48 h with reciprocal shaking at 180 rpm. The data represent the average ± standard deviation of three independent trials. (DOCX) [file pone.0125163.s003.docx]

$V= \frac{4}{3}\pi\cdot\frac{x}{2}\cdot\left( \frac{y}{2} \right)^{2}$

**S1 Table. P(LA-*co*-3HB) production in *E. coli* JM109 and selected transposon mutant.**

| Genotype | Plasmid | Cell dry weight (g/l) | True cell weight (g/l) | Polymer production (g/l) | | |
| --- | --- | --- | --- | --- | --- | --- |
|  |  |  |  | Total | LA | 3HB |
| JM109 (Wild-type) | pTV118N*pct*  *phaC1*_Ps_(ST/QK)*AB* | 6.2 ± 0.5 | 3.3 ± 0.3 | 2.9 ± 0.2 | 0.1 ± 0.1 | 2.8 ± 0.1 |
| JM109 C21  (transposon insertion) | pTV118N*pct*  *phaC1*_Ps_(ST/QK)*AB* | 9.7 ± 1.3 | 4.7 ± 0.1 | 5.1 ± 0.3 | 0.3 ± 0.1 | 4.8 ± 0.3 |

All strains were grown in 1.7 ml of LB medium containing 20 g/l of glucose at 30 °C for 48 h with reciprocal shaking at 180 rpm. The data represent the average ± standard deviation of three independent trials.
