# Supplementary material for: MtgA Deletion-Triggered Cell Enlargement of Escherichia coli for Enhanced Intracellular Polyester Accumulation
Source: PLoS One. 2015 Jun 3;10(6):e0125163. doi: 10.1371/journal.pone.0125163 (PMC4454544; doi:10.1371/journal.pone.0125163)
Supplement: S2 Table — E. coli BW25113 (wild type) and JW3175 (ΔmtgA) harboring pGEMphaC1 Ps(ST/QK)AB [50] were grown on LB medium containing 20 g/l of glucose at 30°C for 48 h with reciprocal shaking at 180 rpm. The data represent the average ± standard deviation of three independent trials. (DOCX) [file pone.0125163.s004.docx]

$V= \frac{4}{3}\pi\cdot\frac{x}{2}\cdot\left( \frac{y}{2} \right)^{2}$

**S2 Table. P(3HB) production in *mtgA*-deleted strains.**

| Genotype | Plasmid | Cell dry weight (g/l) | True cell weight (g/l) | P(3HB) production (g/l) |
| --- | --- | --- | --- | --- |
| Wild type | pGEM*phaC1*_Ps_(ST/QK)*AB* | 9.2 ± 0.2 | 10.5 ± 0.4 | 5.4 ± 0.2 |
| Δ*mtgA* | pGEM*phaC1*_Ps_(ST/QK)*AB* | 8.0 ± 0.7 | 11.7 ± 0.4 | 7.1 ± 0.1 |

*E. coli* BW25113 (wild type) and JW3175 (Δ*mtgA*) harboring pGEM*phaC1*_Ps_(ST/QK)*AB* [50] were grown on LB medium containing 20 g/l of glucose at 30°C for 48 h with reciprocal shaking at 180 rpm. The data represent the average ± standard deviation of three independent trials.
